# Prevalence, types, and determinants of intimate-partner violence among pregnant women in Northern Uganda: a hospital-based cross-sectional study

**DOI:** 10.1186/s12889-025-24465-7

**Published:** 2025-09-24

**Authors:** Jerom Okot, Cinderella Anena, Nelson James Okema, Denis Oryem Amuku, Jerry Enock Otunnu, Beatrice Lamwaka oweka, Ian Daniel Lutara, Francis Watum, Yakobo Nsubuga, Felix Bongomin, Pebalo Francis Pebolo, Simple Ouma

**Affiliations:** 1 Faculty of Medicine, Gulu University, Gulu, Uganda; 2 Department of Public Health, Gulu University, Gulu, Uganda; 3 Department of Medical Microbiology and Immunology, Gulu University, Gulu, Uganda; 4 Department of Internal Medicine, Gulu Regional Referral Hospital, Gulu, Uganda; 5 Department of Sexual and Reproductive Health, Gulu University, Gulu, Uganda; 6 The AIDS Support Organization, Kampala, Uganda

**Keywords:** Gender based violence, Pregnancy, Antenatal care, Northern uganda

## Abstract

**Background:**

Intimate partner violence (IPV) against women is a global health issue, affecting one in three women worldwide. Exposure to IPV during pregnancy poses significant health risks to the mother and her foetus, leading to various complications. This study aimed to determine the prevalence, types, and determinants of IPV among pregnant women in Northern Uganda.

**Methods:**

A cross-sectional study was conducted at Gulu Regional Referral Hospital’s antenatal care clinic from June to August 2023. Data were collected using semi-structured questionnaires in English or Acholi. Participants were selected through systematic random sampling. Information on socio-demographic characteristics, partner attributes, and IPV exposure was collected. Descriptive statistics and modified Poisson regression analyses were performed using STATA 18.0. Associations between variables and IPV were reported as adjusted prevalence ratios (aPR), with *p* < 0.05 considered statistically significant.

**Results:**

Among the 339 participants, the mean age (standard deviation) was 26.1(5.5) years. Overall, 73.2% (*n* = 248) of the participants were exposed to IPV in pregnancy. The most common form of IPV was controlling behaviour by male partners (61.9%, *n* = 210), followed by emotional violence (34.8%, *n* = 118), economic violence (29.5%, *n* = 100), sexual violence (28.9%, *n* = 98), and physical violence (16.2%, *n* = 55). Factors associated with IPV included being in a polygynous marriage (aPR: 1.2, 95% CI: 1.03–1.31, *p* = 0.013), having poor (aPR: 1.6, 95% CI: 1.32–1.89, *p* < 0.0001) or good (aPR: 1.2, 95% CI: 1.03–1.51, *p* = 0.026) versus perfect relationship with the husband’s family, week of amenorrhea (aPR: 1.01, 95% CI: 1.003–1.02, *p* = 0.006), and maternal age (aPR: 0.98, 95% CI: 0.97–0.99, *p* = 0.003).

**Conclusions:**

IPV during pregnancy, particularly controlling behaviour by male partners, is highly prevalent in Northern Uganda. To mitigate the negative impacts on maternal and foetal health, targeted interventions by the Ministry of Health, development partners, and other stakeholders are urgently needed to prevent and manage IPV in pregnancy.

**Supplementary Information:**

The online version contains supplementary material available at 10.1186/s12889-025-24465-7.

## Background

Intimate-partner violence (IPV) remains one of the gravest challenges to women’s health and well-being, it is largely driven by inequity and social injustice [1, 2]. Intimate Partner Violence (IPV) refers to any behaviour within an intimate relationship that causes physical, sexual, or psychological harm to one partner. This includes acts of physical aggression, sexual coercion, psychological abuse, and controlling behaviours. IPV can occur in both public and private settings and affects individuals regardless of gender, although women are disproportionately affected [2, 3].

IPV is one of the systemic public health problems that is increasingly visible on the global health and development agenda [4]. A recent report showed that an estimated 25% of women aged 15 years and above experience at least one form of IPV in their lifetime [5]. World Health Organisation (WHO) revealed that the prevalence of IPV ranged from 15% in urban areas to 71% in rural areas [2]. Further evidence showed that the problem of IPV is most prominent in developing countries, especially in sub-Saharan African (SSA) countries, where socioeconomic status is low, and education is limited [6]. In Northern Uganda, a study in a rural setting showed that women were more likely to get exposed to violence during pregnancy [7].

A primary concern of violence during pregnancy relates to the serious consequences not only for the woman but also for the foetus and ultimately, the child’s growth development [8]. The phenomena have received considerable attention, particularly the adverse consequences of IPV to both the mother and the foetus [9]. Several previous works pointed out several adverse consequences of IPV during pregnancy to the mothers, the foetus, and the newborn baby [9, 10]. The adverse maternal impacts of IPV in pregnancy include maternal mental health problems including anxiety and depression, sexually transmitted infections in case of sexual violence, poor weight gain during pregnancy, increased risk of operative delivery as well as heightened risk of maternal mortality [2, 11]. Meanwhile, the adverse health impacts of IPV in pregnancy include low birth weight, preterm delivery, intrauterine foetal death in case of physical violence, and increased risk of neonatal mortality [12].

Despite its importance, far too little attention has been paid to the epidemiology of IPV during pregnancy in Uganda and there is an increase in the prevalence of violence from 26.7 to 32.3% during pregnancy among women in rural settings [7, 13]. Conversely, a 2020 National Survey on Violence Against Women and Girls (VAWG) found an overall low prevalence of violence during pregnancy, lower than figures reported in the UDHS (2016), however, the survey only explored physical violence and no other forms of IPV [14]. There is regional variation in the prevalence and burden of IPV with Northern Uganda having rates as high as 47% among women attending reproductive health services [14]. The within-country variation is attributable to methodological differences, the working conceptual definition and the measure of the various forms of violence [14, 15]. Besides, the glaring poverty and post-conflict situation in Northern Uganda makes IPV a common place [16]. Focusing on the understanding and epidemiology of IPV during pregnancy will help in designing interventions to curb the negative effect on the mothers and their unborn babies [17]. This study aims to address this important but under-researched issue by investigating IPV among pregnant women in Northern Uganda. The findings could provide valuable insights for stakeholders on the prevalence, types and factors associated with IPV in this population and guide the development of context-specific and culturally appropriate interventions to prevent and manage IPV. Ultimately, this research aimed to determine the prevalence, types, and determinants of IPV among pregnant women in Northern Uganda.

## Methods

### Study design and setting

This facility-based cross-sectional study was conducted at the ANC clinic at Gulu Regional Referral Hospital (GRRH). GRRH is a tertiary hospital for patients from mid-northern Uganda, thus serving both rural and urban populations within the catchment area. GRRH also receives patients from neighbouring countries of South Sudan and the Democratic Republic of Congo. It is also a teaching hospital for the Gulu University, Trainer Centre for Fellowship with East Central and Southern African College of Obstetrics and Gynaecology (ECSACOG) and many other Diploma and Certificate Training Institutions in the region. It is a hospital with both out and in-patient services for an estimated 120,000 patients per year. The hospital has specialised units managed by consultants from GRRH and the Faculty of Medicine, at Gulu University. The Obstetrics and Gynaecology department provides all services ranging from ANC to Postnatal Care (PNC) during pregnancy and after delivery. Due to the quality and free ANC services, a large proportion of pregnant women within the catchment area attend their ANC services at GRRH.

### Study population

This study was conducted among pregnant women attending ANC at GRRH. All pregnant women attending ANC at GRRH were eligible for inclusion, irrespective of gestational age. However, we set to exclude any pregnant woman who would be deemed incompetent to consent due to severe sickness or mental disorder, but none were excluded.

### Sample size

Using the Kish Leslie formula, the prevalence of IPV in pregnancy was 26.7% from a study conducted at two rural health centres in Northern Uganda [18], and at a 5% significance, the minimum sample size was estimated to be 301. Assuming a conservative non-response rate of 10% (301/0.9), the adjusted sample size was 335 participants.

### Sampling techniques

Based on the average daily attendance at the ANC clinic at GRRH which 80 women per day was approximately, we used a systematic random sampling method aided by the daily ANC attendance register to select every 5th person on the list on any given day. While on the ground the daily ANC attendance ranged between 60 and 100 pregnant women and thus, we collected data from between 12 and 20 participants on any given day.

### Data collection

Data were collected from 339 pregnant women attending their ANC at GRRH between June and August 2023. Data were collected using a semi-structured questionnaire which was developed for this study by the authors. Originally, the tool was developed in English and later translated into Acholi language (Luo) and then pre-tested, for consistency in question interpretation and language appropriateness, among 20 pregnant women attending ANC at Laroo Health Centre III. Data were collected using interviewer-administered questionnaires in either Acholi or English language depending on the participant’s literacy level and preferences.

### Study variables

Independent variables included socio-demographic characteristics like age in years recorded based on self-reported date of birth, education which was assessed via a single question asking about the highest level, being in a union which was either married or cohabiting, employment status which was assessed by asking participants whether they were currently engaged in any form of paid work, partner level of education, partner employment status, age at first sex, age at first marriage, age of the pregnancy in weeks and, living with HIV and partner alcohol use. The outcome variable for this study was the experience of IPV. The participants were asked batteries of questions to identify whether they were exposed to any of the various forms of violence notably physical violence, emotional violence, economic violence, sexual violence or controlling behaviours. Our tool for screening for IPV was adapted from questionnaires previously used to screen IPV among women in the same setting, as detailed in Table 2, a positive response to any item within a given category was considered indicative of experiencing that form of IPV.

### Data management and statistical analysis

Data were entered and cleaned in Microsoft Excel 2023 and then exported to STATA 18.0 for analysis. Descriptive statistics were summarised using frequency with corresponding proportions for categorical variables or mean with corresponding standard deviations (SD) for continuous variables. To examine the associations between independent factors and experiencing any form of IPV, we conducted univariate and multivariable modified Poisson regression analyses with robust standard errors. We reported univariate analyses using unadjusted prevalence ratios (uPR) with corresponding confidence intervals (CI) and p-value.

All independent variables with *p* < 0.20 at univariable analysis were considered for inclusion into the multivariable modified Poisson regression model. Since they have a study power of greater than 80%, final model selection was guided by the Akaike information criterion (AIC), which balances model fit and complexity. As a result some variable with *p* < 0.2 were excluded from the final model due to their limited contribution to over model performance. The results from the multivariable model were reported using the adjusted Prevalence ratios (aPR) with corresponding 95% CIs and p-values. Any *p* < 0.05 or effect size whose CI did not include the null value (0) was considered statistically significant.

## Results

### Baseline characteristics of the participants

A total of 339 pregnant women were included in this study. By trimester, the majority (43.6%, *n* = 136) were in the second trimester, followed by the third trimester (37.5%, *n* = 117) and first trimester (18.9%, *n* = 59). The mean age (SD) was 26.1(5.5) years, the mean age (SD) at first sex was 17.8 (2.5) years and the mean age (SD) at first marriage was 20.3 (3.3) years. More than a third of the participants attained an ordinary level of education (33.6%, *n* = 113), and 28.0%(*n* = 94) and 20.62%(*n* = 68) attained an upper primary and tertiary level of education respectively. Most of the participants (89.7%, *n* = 304) were in union (married or cohabiting), were in a monogamous marriage (75.5%, *n* = 253) and employed (46%, *n* = 156). Furthermore, 38.5% (*n* = 130) of their partners attained a tertiary level of education, and 5.6%(*n* = 19) had a lower primary or no education. About half (56.7%; *n* = 190) of the participants ever used contraceptives, 8.3%(*n* = 28) were living with HIV and 42.4%(*n* = 143) of their partners take alcohol. The mean gestation age (SD; range) was 23.2 (9.0; 4–38) weeks, and the majority (88.3% (*n* = 294)) of respondents conceived of the desire of both husband and wife (Table 1).

**Table 1:** Baseline characteristics of study participants by IPV during pregnancy

Characteristics	All (*N* = 339) *n* (%)
Age in years (mean, SD)	26.08(5.51)
Place of residence	
Gulu Another district	288(89.4) 34(10.6)
The education level of the participant	
Lower primary or none Upper Primary Secondary Tertiary	35(10.4) 94(28.1) 139(41.3) 68(20.2)
Marital status	
Not in union In union	35(10.3) 304(89.7)
HIV status	
Negative Positive	311(91.7) 28(8.3)
Has a co-wife	
No Yes	253(75.5) 82(24.5)
Employment status	
Not employed Employed	183(54) 156(46)
Employment status of partner	
Not employed Employed	67(19.8) 271(80.2)
Education level of partner	
Lower Primary Upper Primary O level A level Tertiary	19(5.6) 29(8.6) 99(29.3) 61(18) 130(38.5)
Ever used contraceptive	
No Yes	145(43.3) 190(56.7)
Was this pregnancy intended?	
No Yes	115(33.9) 224(66.1)
Pregnancy wanted by husband	
Husband or both Wife alone	294(88.3) 39(11.7)
Marriage type	
Married the husband she loves Arranged marriage	255(81.5) 58(18.5)
Relationship with husband’s family	
Very good Good Poor or fair	100(29.9) 145(43.3) 90(26.9)
Husband drink alcohol	
No Yes	194(57.6) 143(42.4)
Quartiles of income	
Lower quartile Second quartile Third quartile Highest quartile	46(25.5) 68(27.1) 61(24.3) 58(23.1)
Quartile of income of partner	
Lowest quartile Second quartile Third quartile Highest quartile	64(25.5) 68(27.1) 61(24.3) 58(23.1)
Age (years) at first sexual intercourse (mean, SD)	17.79(2.46)
Age (years) of first marriage (mean, SD)	20.32(3.30)
Number of children at home (mean, SD)	1.61(1.36)
Week of amenorrhea (mean, SD)	23.19(8.99)

### Prevalence and types of IPV in pregnancy among women in Northern Uganda

Almost three in every four (73.2%) pregnant women in Northern Uganda experienced at least one form of IPV during the current pregnancy. The most common form of IPV in pregnancy was controlling behaviours by the male partner (61.9%, *n* = 210), followed by emotional violence (34.8%, *n* = 118), economic violence (29.5%, *n* = 100), and sexual violence (28.9%, *n* = 98). Meanwhile, physical violence (16.2%, *n* = 55) was the least common form of violence during pregnancy (Table 2).

**Table 2:** Prevalence and types of IPV in pregnancy among women in Northern Uganda

Different forms of violence	Exposed to violence	
	*n*/*N*	(%)
Exposure to physical violence		
Slapped or had something thrown at her that could hurt her	46/338	(13.6%)
Was pushed or shoved	22/339	(6.5%)
Was hit with a fist or something else could hurt	14/338	(4.1%)
Was choked or burnt on purpose	10/337	(3.0%)
Threatened or hurt by a gun, knife, or other weapon	6/339	(1.8%)
Was punched or kicked in the abdomen while pregnant	5/338	(1.5%)
Exposed to at least one form of physical violence	**55/339**	**(16.2%)**
Exposure to sexual violence		
Physically forced to have sexual intercourse	77/339	(22.7%)
Forced into sexual intercourse	74/339	(21.8%)
Forced into sexual acts she found degrading or humiliating	25/339	(7.4%)
Exposed to at least one form of sexual violence	98/339	(28.9%)
Exposure to emotional violence		
Insulted or made feel bad about herself	95/336	(28.3%)
Belittled or humiliated in front of others	47/339	(13.9%)
Scared or intimidated on purpose by looking or yelling at or smashing things	51/338	(15.1%)
Threat to hurt someone you cared about	18/339	(5.3%)
Exposed to at least one form of emotional violence	**118/339**	**(34.8%)**
Exposure to economic violence		
Destroyed your property intentionally	23/339	(6.8%)
Restricted your access to financial resources	43/339	(12.7%)
Restricted you from working	45/339	(13.3%)
Restricted you from studying	26/339	(7.7%)
Did not provide for your pregnancy need	44/339	(13.0%)
Exposed to at least one form of economic violence	**100/339**	**(29.5%)**
Exposed to controlling behaviours		
Tried to keep you from seeing your friends	111/339	(32.7%)
Tried to restrict contact with your family of birth	60/337	(17.8%)
Insisted on knowing where she always	106/339	(31.3%)
Ignored you and treated you indifferently	66/339	(19.5%)
Got angry if you speak with other men	112/339	(33.0%)
Suspicious that you were unfaithful	61/339	(18.0%)
Expected you to ask permission before seeking healthcare	36/339	(10.6%)
Exposed to controlling behaviours as a form of IPV	**210/339**	**(61.9%)**
Exposed to any IPV	**248**	**(73.2%)**

### Determinants of IPV in pregnancy among women in Northern Uganda

At the univariate level, factors associated with IPV in pregnancy were being in a polygyny marriage (uPR: 1.2, 95% CI: 1.07–1.36, *p* = 0.002), partner not employed (uPR: 1.2: 95% CI: 1.04–1.35, *p* = 0.009), pregnancy being unwanted by either or both partner (uPR: 1.3, 95% CI: 0.85–1.10, *p* < 0.0001), having poor (uPR: 1.6, 95% CI: 1.36–1.94, *p* < 0.0001) or good (uPR: 1.3, 95% CI: 1.03–1.53, *p* = 0.022) versus perfect relationship with the husband’s family, week of amenorrhea (uPR: 1.01, 95% CI: 1.003–1.02, *p* = 0.008), participant’s age (cPR: 0.98, 95% CI: 0.96–0.99, *p* = 0.002), participant’s age at first sex (uPR: 0.95, 95% CI: 0.92–0.97, *p* < 0.0001), and participant’s age at first marriage (uPR: 0.97, 95% CI:0.95–0.99, *p* = 0.005) (Table 3).

**Table 3:** Factors associated with exposure to IPV in pregnancy

Variable	IPV		Unadjusted PR (95% CI)	*P*-value
	No (n = 91) *n* (%)	Yes (n = 248) *n* (%)		
Age in completed years, mean (SD)	27.65(5.48)	25.50(5.41)	0.98(0.96–0.99)	0.002
Level of education				
Lower primary or none Upper Primary Secondary school Tertiary	11(12.1) 20(22.0) 28(30.8) 32(35.2)	24(9.8) 74(30.2) 111(44.6) 36(15.3)	Reference 1.1(0.89–1.44) 1.1(0.91–1.45) 0.8(0.58–1.06)	0.296 0.239 0.114
Married/cohabiting				
Not in union In union	6(6.6) 85(93.4)	29(11.7) 219(88.3)	Reference 0.9(0.74–1.03)	0.102
HIV status				
Not living with HIV Living with HIV	6(6.6) 85(93.4)	29(11.7) 219(88.3)	Reference 0.9(0.78–1.24)	0.918
Husband has other wife or wives				
No Yes	77(86.5) 12(13.5)	176(71.5) 70(28.5)	Reference 1.2(0.07-1.36)	0.002
Employment status				
Not employed Employed	44(48.4) 47(51.6)	139(56) 109(44)	1.02(0.94–1.22) Reference	0.302
Husband employment status				
Not employed Employed	10(11.1) 80(88.9)	57(23) 191(77)	1.2(1.04–1.35) Reference	0.009
Partner’s level of education				
Lower primary or less Upper Primary Tertiary	40(44) 6(6.6) 45(49.5)	111(44.6) 23(9.2) 115(46.2)	Reference 1.1(0.87–1.33) 0.9(0.85–1.12)	0.477 0.747
Ever used contraceptive				
No Yes	24(26.4) 67(73.6)	91(36.7) 157(63.3)	Reference 0.9(0.85–1.10)	0.642
Partner wanted the pregnancy				
Both wanted Only one or none	85(93.4) 6(6.6)	209(86.4) 33(13.6)	Reference 1.3(1.16–1.46)	< 0.0001
Relationship with husband’s family				
Perfect Good Poor	43(48.3) 41(46.1) 5(5.6)	57(23.2) 104(42.3) 85(34.6)	Reference 1.3(1.03–1.53) 1.6(1.36–1.94)	0.022 < 0.0001
Age at first sex	18.72(2.40)	17.45(2.39)	0.95(0.92–0.97)	< 0.0001
Age at first marriage	21.27(3.69)	19.98(3.09)	0.97(0.95–0.99)	0.005
Week of amenorrhea, mean (SD)	21.16(9.40)	23.91(8.75)	1.01(1.003–1.02)	0.008
Number of children, mean (SD)	1.72(1.30)	1.57(1.38)	0.97(0.92–1.02)	0.177

After adjusting for being in a union, being in a polygynous marriage (aPR: 1.2, 95% CI: 1.03–1.31, *p* = 0.013), having poor (aPR: 1.6, 95% CI: 1.32–1.89, *p* < 0.0001) or good (aPR: 1.2, 95% CI: 1.03–1.51, *p* = 0.026) versus perfect relationship with the husband’s family, week of amenorrhea (aPR: 1.01, 95% CI: 1.003–1.02, *p* = 0.006), and maternal age (aPR: 0.98, 95% CI: 0.97–0.99, *p* = 0.003), all remained independently associated with IPV in pregnancy (Table 4).

**Table 4:** Factors independently associated with IPV in pregnancy

Variable	Adjusted PR (95% CI)	*P* value
Married/cohabiting		
No Yes	Reference 1.04(0.89–1.21)	0.600
Husband has other wife or wives		
No Yes	Reference 1.2(1.03–1.31)	0.013
Relationship with husband’s family		
Perfect Good Poor	Reference e 1.2(1.03–1.51) 1.6(1.32–1.89)	0.026 < 0.0001
How old is the pregnancy in weeks	1.01(1.003–1.02)	0.006
Age in completed years	0.98(0.97–0.99)	0.003

## Discussion

Intimate partner violence (IPV) is a widespread public health concern affecting women globally. IPV during pregnancy presents serious health risks for both mother and foetus.

This study found an alarming overall prevalence of IPV during pregnancy of 73.2%. The most common forms were controlling behaviours (61.9%), followed by emotional violence (34.8%), economic violence (29.5%), sexual violence (28.9%,), and physical violence (16.2%).

This finding is similar to that from a study done in South West (70.3%) [4], however notably higher than figures in other regions of Uganda, 40.6% among teenage mothers in Lira district [7] and 27.8% in eastern Uganda [13]. As well as other countries such as Malawi and Sri Lanka 38.6% and 39.7% respectively [10, 19]. The high IPV rate in this study may be partially attributed to the inclusion of controlling behaviours in the IPV definition, which have not been measured in other studies. Such methodology difference should be considered when interpreting the prevalence comparisons. Male controlling behaviour targeting women may be attributed to unequal gender norms of patriarchy of male dominance and its worst forms using social tolerance and acceptance of violence. Addressing IPV in pregnancy requires a comprehensive approach that encompasses prevention, intervention, and support services. Primary prevention efforts should focus on addressing underlying social norms and attitudes that perpetuate IPV, such as gender inequality. Providing education and awareness programs for both men and women can help challenge these norms and promote healthy relationships. In addition, healthcare provider plays a critical role in identifying and addressing IPV during pregnancy. Routine screening for IPV should be emphasised during prenatal care services allowing identification of individuals at risk and provision of appropriate support and referrals. Training healthcare providers on how to sensitively inquire about IPV and respond to disclosures is essential for ensuring effective intervention.

Women in polygamous marriages had 1.2 times the prevalence of IPV in pregnancy compared to women in monogamous marriages. This finding is in agreement with a report from Lira District, Northern Uganda showing that women in a polygamy marriage were more likely to get exposed to intimate partner violence (*p* = 0.001) [7]. A study in Sub-Saharan Africa [20], showed that polygyny is associated with intimate partner violence among the general population. Polygyny makes IPV worse during pregnancy because of unfair power differences, society’s beliefs and women not having enough to say. Jealousy among wives, not getting enough care and pressure to follow traditional roles can also lead to emotional or physical harm.

This study also showed that women who had poor or good relationships with their husband’s family members had 1.6 and 1.2 times the prevalence of IPV in pregnancy compared to women who had perfect relationships with their husbands’ family members. Associations between sub-optimal relationships with husband’s relatives and women’s exposure to IPV was established in this study. Having poor or sub-optimal relationships with the husband’s family members compared with having perfect relationships with the husband’s family members were all positively associated with exposure to IPV among women largely due to family interference by the in-laws [21–23].

There was a 2% reduction in the prevalence of IPV for every one-year increase in age of the pregnant woman. An increase in age, therefore, could potentially have a protective effect on IPV, this finding was in line with findings from conflict-affected areas in northern Uganda [24] and elsewhere [25–27], reporting that younger age women were more exposed to IPV. There was a 2% decrease in the risk of exposure to IPV in pregnancy for every one-year increase in the age of the mother. Moreover, younger women less than 25.5 years old could be less mature emotionally to navigate relationship dynamics and are generally vulnerable. These results suggest that the younger mothers often experience stressful transitions to parenthood which can trigger violence compared to older mothers.

We also found that, for every one-week increase in gestational age, there is a 1% increase in the prevalence of IPV in pregnancy. To the best of our knowledge, there has been no previous report of similar findings. We hypothesise that as pregnancy progresses, the prevalence of IPV in pregnancy might increase due to cumulative time on the risk of IPV in pregnancy. However, there is a possibility that as pregnancy grows pregnancy-related hormonal changes as well as changes in women’s demand might be the mechanisms through which IPV in pregnancy increases with an increase in gestational age [28]. It is worth noting that IPV can occur at any stage of pregnancy, therefore midwives and obstetricians should endeavour to screen every pregnant woman for IPV in pregnancy and providing comprehensive support services remains essential to ensure the safety and well-being of pregnant individuals.

### Limitations of the study

The findings from this study were self-reported and. this being a hospital-based rather than population-based study makes generalizability impossible. In addition, men were not involved, therefore it’s not possible to determine risk factors and reasons for the perpetration of IPV.

## Conclusion

IPV during pregnancy, particularly controlling behaviour by male partners, is highly prevalent in Northern Uganda. Younger women, women in polygamy marriages and those having poor or sub-optimal relationships with the husband’s family members were most affected. To mitigate the negative impacts on maternal and foetal health, targeted interventions by the Ministry of Health, development partners, and other stakeholders are urgently needed to prevent and manage IPV in pregnancy.

## Supplementary Information

Below is the link to the electronic supplementary material.

Supplementary Material 1

Supplementary Material 2

## Data Availability

All relevant data are within the manuscript and its supporting information files. Questionnaire has been attached as supplementary file Data are available upon reasonable request from the first author.

## Abbreviations

ANC: Ante-natal care
ECSACOG: East Central and Southern African college of obstetrics and gynaecology
IPV: Gender based violence
GUREC: Gulu research ethical committee
GRRH: Gulu regional referral hospital
PNC: Post-natal care
PR: Prevalence ratio SSA: Sub-Saharan Africa
SD: Standard deviation
VAW: Violence against women and girls
WHO: World health organisation
