# Supplementary material for: Prevalence, types, and determinants of intimate-partner violence among pregnant women in Northern Uganda: a hospital-based cross-sectional study
Source: BMC Public Health. 2025 Sep 24;25:3059. doi: 10.1186/s12889-025-24465-7 (PMC12462169; doi:10.1186/s12889-025-24465-7)
Supplement: Supplementary file 1 — Supplementary Material 1 [file 12889_2025_24465_MOESM1_ESM.docx]

**Important information. Please read.**

- This form should be used by authors to request any change in authorship (adding/deleting authors) including changes in corresponding authors. This form should not be used for name changes. Please fully complete all sections. Use black ink and block capitals and provide each author’s full name with the given name first followed by the family name.

- By signing this declaration, all authors guarantee that the order of the authors are in accordance with their scientific contribution, if applicable as different conventions apply per discipline, and that only authors have been added who made a meaningful contribution to the work.

- Please note, in author collaborations where there is formal agreement for representing the collaboration, it is sufficient for the representative or legal guarantor (usually the corresponding author) to complete and sign the Authorship Change Form on behalf of all authors, **next to the added/removed author(s)**. **(Complete Section 3 followed by Section**

**6.)**

In author collaborations where there is no formal agreement for representing the collaboration and **there are more than 10 authors**, one may sign for all, provided the signer appends correspondence that attests that each of the authors have agreed to the change **and the added/removed authors sign the form**. **(Complete Section 3 followed by Section**

**6.)**

- Please note, we cannot investigate or mediate any authorship disputes. If you are unable to obtain agreement from all authors (including those who you wish to be removed) you must refer the matter to your institution(s) for investigation. Please inform us if you need to do this.

- If you are not able to return a fully completed form within **30 days** of the date that it was sent to the author requesting the change, we may have to withdraw your manuscript. We cannot publish manuscripts where authorship has not been agreed by all authors (including those who have been removed).

- Incomplete forms will be rejected.

- Please return/upload this form, fully completed, to the Journals Editorial Office. The Journal and/or Publisher will consider the information you have provided to decide whether to approve the proposed change in authorship. We may decide to contact your institution for more information or undertake a further investigation, if appropriate, before making a final decision.

| **Manuscript ID no.:** 0e531b4a-2b94-4154-bdcb-5b8cf3e44a1c v3.0 |
| --- |

**Section 1: Please provide the current title of manuscript**

Title: Prevalence, types, and Determinants of Intimate-partner Violence among Pregnant Women in Northern Uganda: A Hospital-based Cross-sectional study

**Section 2: Please provide the previous authorship, in the order shown on the manuscript before the changes were introduced. Please indicate the corresponding author by adding (CA) behind the name.**

|  | First name(s) | Family name | ORCID or SCOPUS id, if available |
| --- | --- | --- | --- |
| 1^st^ author | Jerom | Okot (CA) |  |
| 2^nd^ author | Cinderella | Anena |  |
| 3^rd^ author | Nelson James | Okema |  |
| 4^th^ author | Denis | Oryem Amuku |  |
| 5^th^ author | Jerry Enock | Otunnu |  |
| 6^th^ author | Beatrice | Lamwaka Oweka |  |
| 7^th^ author | Ian Daniel | Lutara |  |
| 8^th^ author | Yakobo | Nsubuga |  |
| 9^th^ author | Felix | Bongomin |  |
| 10^th^ author | Pebalo Francis | Pebolo |  |
| 11^th^ author | Simple | Ouma |  |

Please use an additional sheet if there are more than 10 authors.

**Section 3: Please provide a justification for change. Please use this section to explain your reasons for changing the authorship of your manuscript, e.g. what necessitated the change in authorship? Please refer to the (journal) policy pages for more information about authorship. Please explain why omitted authors were not originally included and/or why authors were removed on the submitted manuscript.**

| Francis Watum played a critical role in drafting key sections of the manuscript and actively participated in reviewing and approving the final version. His involvement meets the journal's criteria for authorship, and we believe it is both necessary and appropriate to acknowledge his intellectual contribution to the work. No authors have been removed from the original list. |
| --- |

**Section 4: Proposed new authorship. Please provide your new authorship list in the order you would like it to appear on the manuscript. Please indicate the corresponding author by adding (CA) behind the name. If the Corresponding Author has changed, please indicate the reason under section 3.**

|  | First name(s) | Family name (this name will appear in full on the final publication and will be searchable in various abstract and indexing databases) | Affiliated institute | E-mail address |
| --- | --- | --- | --- | --- |
| 1^st^ author | Jerom | Okot (CA) | Gulu University | [okotayolijerome@gmail.com](mailto:okotayolijerome@gmail.com) |
| 2^nd^ author | Cinderella | Anena | Gulu University | [cinderellaanena1@gmail.com](mailto:cinderellaanena1@gmail.com) |
| 3^rd^ author | Nelson James | Okema | Gulu University | [ojamesnelson@gmail.com](mailto:ojamesnelson@gmail.com) |
| 4^th^ author | Denis | Oryem Amuku | Gulu University | [denisamuku123@gmail.com](mailto:denisamuku123@gmail.com) |
| 5^th^ author | Jerry Enock | Otunnu | Gulu University | [otunnujerryenock@gmail.com](mailto:otunnujerryenock@gmail.com) |
| 6^th^ author | Beatrice | Lamwaka Oweka | Gulu University | [lamwakabeatrice12@gmail.com](mailto:lamwakabeatrice12@gmail.com) |
| 7^th^ author | Ian Daniel | Lutara | Gulu University | [Iandanlutara@gmail.com](mailto:Iandanlutara@gmail.com) |
| 8^th^ author | Francis | Watum | Gulu University | [watumf@gmail.com](mailto:watumf@gmail.com) |
| 9^th^ author | Yakobo | Nsubuga | Gulu University | [doctornsubuga@gmail.com](mailto:doctornsubuga@gmail.com) |
| 10^th^ author | Felix | Bongomin | Gulu University | [drbongomin@gmail.com](mailto:drbongomin@gmail.com) |
| 11^th^ author | Pebalo Francis | Pebolo | Gulu University | [pebalopebolo@gmail.com](mailto:pebalopebolo@gmail.com) |
| 12^th^ author | Simple | Ouma | Gulu University | [oumasimple@gmail.com](mailto:oumasimple@gmail.com) |

Please use an additional sheet if there are more than 10 authors.

**Section 5: Author contribution, Acknowledgement and Disclosures. Please use this section to provide a new disclosure statement and, if appropriate, acknowledge any contributors who have been removed as authors and ensure you state what contribution any new authors made (if applicable per the journal or book (series) policy). Please ensure these are updated in your manuscript - after approval of the change(s) - as our production department will not transfer the information in this form to your manuscript.**

**New acknowledgements:**

We extend our deepest gratitude to our study participants; whose invaluable contribution and dedication made this research possible. We extend our deepest gratitude to our study participants; whose invaluable contributions and dedications made this research possible. Their willingness to share their time, experience and insights was crucial to the success of the work

**New Disclosures (financial and non-financial interests, funding):**

The authors declare that there is no conflict of interest.

**New Author Contributions statement (if applicable per the journal policy):**

JO, JNO, BL, DOA, JEO, BL, IDL, YN, and SO conceptualised the study, and participated in funds acquisition, JO, JNO, IDL and JEO collected data, and JO and SO conducted data analysis and visualization. JO, SO, FW and CA drafted the manuscript. FB and PFP funding acquisition. ALL authors contributed to the review and approval of the final manuscript

State ‘Not applicable’ if there are no new authors.

**Section 6: Declaration of agreement. *All* authors, unchanged, new and removed *must* sign this declaration.**

(NB: Please print the form, (docu)-sign and return/upload a scanned copy. Please note that signatures that have been inserted as an image file are acceptable as long as it is handwritten. Typed names in the signature box are unacceptable.) *** Please delete as appropriate. Delete all of the bold if you were on the original authorship list and are remaining as an author.**

|  | First name | Family name |  | Signature | Date |
| --- | --- | --- | --- | --- | --- |
| 1^st^ author | Jerom | Okot (CA) | I agree to the proposed new authorship shown in section 4 /**and the addition/removal*****of my name to the authorship list** /and the proposed change in corresponding author | 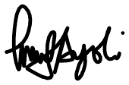 | 12/07/2025 |
| 2^nd^ author | Cinderella | Anena | I agree to the proposed new authorship shown in section 4 /**and the addition/removal*****of my name to the authorship list** /and the proposed change in corresponding author | 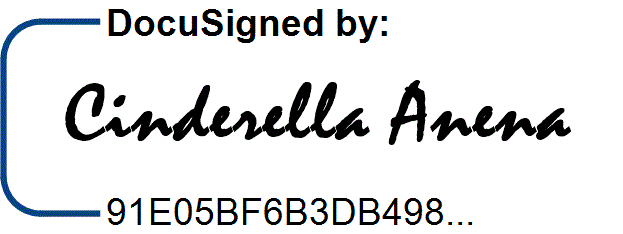 | 2/07/2025 |
| 3^rd^ author | Nelson James | Okema | I agree to the proposed new authorship shown in section 4 /**and the addition/removal*****of my name to the authorship list** /and the proposed change in corresponding author | 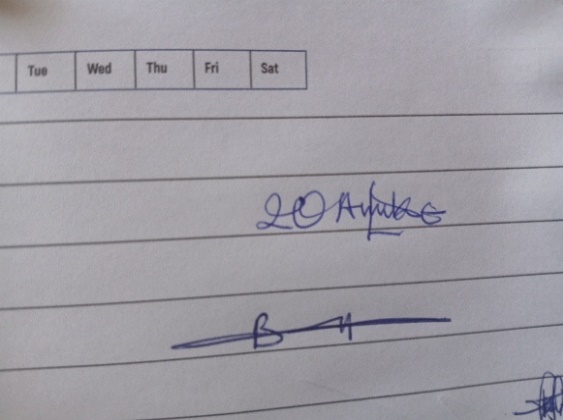 | 12/07/2025 |
| 4^th^ authors | Denis | Oryem Amuku | I agree to the proposed new authorship shown in section 4 /**and the addition/removal*****of my name to the authorship list** /and the proposed change in corresponding author | 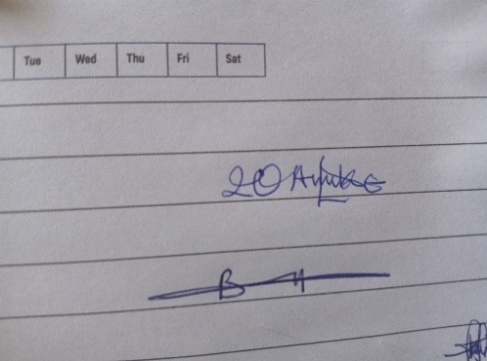 | 12/07/2025 |
| 5^th^ author | Jerry Enock | Otunnu | I agree to the proposed new authorship shown in section 4 /**and the addition/removal*****of my name to the authorship list** /and the proposed change in corresponding author | 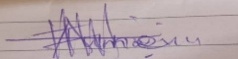 | 12/07/2025 |
| 6^th^ author | Beatrice | Lamwaka Oweka | I agree to the proposed new authorship shown in section 4 /**and the addition/removal*****of my name to the authorship list** /and the proposed change in corresponding author | 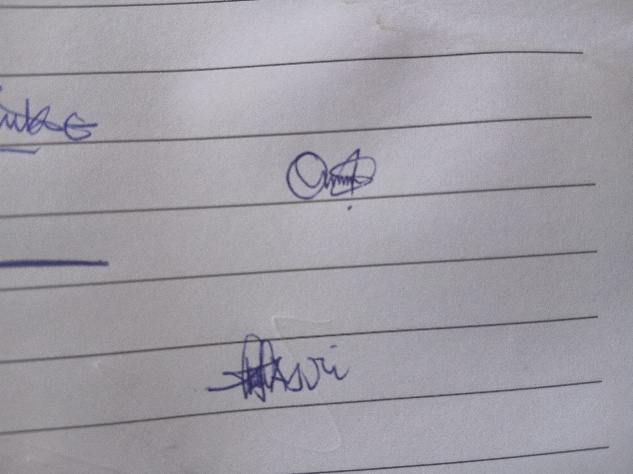 | 12/07/2025 |
| 7^th^ author | Ian Daniel | Lutara | I agree to the proposed new authorship shown in section 4 /**and the addition/removal*****of my name to the authorship list** /and the proposed change in corresponding author | 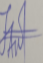 | 12/07/2025 |
| 8^th^ author | Francis | Watum | I agree to the proposed new authorship shown in section 4 /**and the addition/removal*****of my name to the authorship list** /and the proposed change in corresponding author | 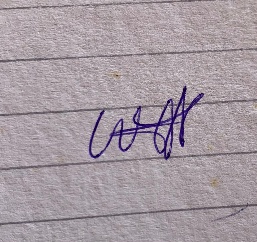 | 12/07/2025 |
| 9^th^ author | Yakobo | Nsubuga | I agree to the proposed new authorship shown in section 4 /**and the addition/removal*****of my name to the authorship list** /and the proposed change in corresponding author | 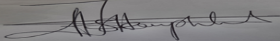 | 12/07/2025 |
| 10^th^ author | Felix | Bongomin | I agree to the proposed new authorship shown in section 4 /**and the addition/removal*****of my name to the authorship list** /and the proposed change in corresponding author | 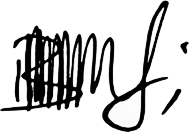 | 12/07/2025 |
| 11^th^ author | Pebalo Francis | Pebolo | I agree to the proposed new authorship shown in section 4 /**and the addition/removal*****of my name to the authorship list** /and the proposed change in corresponding author |  | 12/07/2025 |
| 12^th^ author | Simple | Ouma | I agree to the proposed new authorship shown in section 4 /**and the addition/removal*****of my name to the authorship list** /and the proposed change in corresponding author | 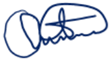 | 12/07/2025 |

Please use an additional sheet if there are more than 10 authors.

**Both added/removed authors should complete the information in the first table under Section 6.**

---- End of form ----
