# Supplementary material for: Prevalence, types, and determinants of intimate-partner violence among pregnant women in Northern Uganda: a hospital-based cross-sectional study
Source: BMC Public Health. 2025 Sep 24;25:3059. doi: 10.1186/s12889-025-24465-7 (PMC12462169; doi:10.1186/s12889-025-24465-7)
Supplement: Supplementary file 2 — Supplementary Material 2 [file 12889_2025_24465_MOESM2_ESM.docx]

**Data collection tool**

**Date of interview**……………………………………….

**Section A: Socio-demographic characteristics of the participants**

1. Age (in completed years)

-----------------------------------------------

1. What is your highest level of education?
2. None
3. Lower Primary
4. Upper Primary
5. O-level
6. A-level
7. Tertiary
8. Where do you stay?

Village………………………………

Parish……………………………….

Sub-county…………………………

County…………………………….

District…………………………….

1. What is your marital status?
2. Never married.
3. In a union (married or cohabiting)
4. Divorced
5. Windowed
6. At what age did you get married?

…………………………………………………………

1. At what age did you first have sex?

…………………………………………………………

1. What type of marriage?
2. Love marriage.
3. Arranged married.
4. What is your employment status?
5. Employed
6. Unemployed
7. If employed, what is your occupation?

…………………………………………………………………………

1. What is your estimated monthly income?

…………………………………………………………………Ugandan shillings

1. When was your last normal menstrual period? (check ANC card to confirm)

……………………………………………………………………………..

1. Was this current pregnancy intended?
2. Yes
3. No
4. Did both of you (you and your husband) want the pregnancy?
5. Yes
6. Only me
7. Only the man
8. What is your partner’s level of education?
9. None
10. Lower Primary
11. Upper Primary
12. O level
13. A level
14. Tertiary
15. What is your husband's employment status?
16. Employed
17. Unemployed
18. What is your husband's estimated monthly income?

……………………………………………………………………Ugandan shillings

1. Does your husband have other wives or wives?
2. Yes
3. No
4. How do you rate the relationship between you and your husband`s family?
5. Very good
6. Good
7. Fair
8. Poor
9. Very poor
10. How many children do you have? …………………………………………………..
11. Have you ever used modern contraception?
12. Yes
13. No
14. What is your HIV status?
15. Negative
16. Positive
17. Unknown
18. Does your husband take alcohol?
19. Yes
20. No

**Section B: Gender-based violence screening tool** (*Please respond with a No/Yes answer*)

| No | Question | Current pregnancy | Past pregnancy | When not pregnant |
| --- | --- | --- | --- | --- |
| **Physical violence by an intimate partner** | | | | |
| 1 | Was slapped or had something thrown at her that could hurt her. |  |  |  |
| 2 | Was pushed or shoved. |  |  |  |
| 3 | Was hit with a fist or something else could hurt. |  |  |  |
| 4 | Was choked or burnt on purpose |  |  |  |
| 5 | The perpetrator threatened to use or used a gun, knife, or other weapons against her |  |  |  |
| 6 | Was punched or kicked in the abdomen while pregnant |  |  |  |
| 7 | If yes to any of the above, was violence less, the same, or better than before the current pregnancy? |  |  |  |
| **Sexual violence by an intimate partner** | | | | |
| 8 | Was physically forced to have sexual intercourse when she did not want to |  |  |  |
| 9 | Had sexual intercourse when she did not want to because she was afraid of what her partner might do |  |  |  |
| 10 | Was forced to do something sexual that she found degrading or humiliating |  |  |  |
| **Emotional violence by an intimate partner** | | | | |
| 11 | Was insulted or made to feel bad about herself |  |  |  |
| 12 | Was belittled or humiliated in front of other people |  |  |  |
| 13 | The perpetrator had done things to scare or intimidate her on purpose by the way he looked at her by yelling or smashing things |  |  |  |
| 14 | The perpetrator had threatened to hurt someone she cared about |  |  |  |
| **Economic violence** | | | | |
| 15 | Destroyed my property intentionally |  |  |  |
| 16 | Restricted me from accessing financial resources |  |  |  |
| 17 | Restricted me from working |  |  |  |
| 18 | Restricted me from studying |  |  |  |
| 19 | Did not provide for me or pregnancy need |  |  |  |
| **Controlling behaviours by an intimate partner** | | | | |
| 20 | He tried to keep her from seeing friends |  |  |  |
| 21 | He tried to restrict contact with her family of birth |  |  |  |
| 22 | He insisted on knowing where she was always |  |  |  |
| 23 | He ignored her and treated her indifferently |  |  |  |
| 24 | He got angry if she spoke with another man |  |  |  |
| 25 | He was often suspected that she was unfaithful |  |  |  |
| 26 | He expected her to ask permission before seeking healthcare |  |  |  |

**Section C: Maternal major depression assessment**

| **No** | **Question** | No | Yes |
| --- | --- | --- | --- |
| 1 | For the past weeks, were you depressed or down, or felt sad, empty, or hopeless most of the day, nearly every day? |  |  |
| 2 | In the past two weeks, were you much less interested in most things or much less able to enjoy the things you used to enjoy, most of the time? |  |  |
|  | **Over the two weeks, when you felt depressed or uninterested:** |  |  |
| 3. | Was your appetite decreased or increased nearly every day? Did your weight decrease or increase without trying intentionally? |  |  |
| 4. | Did you have trouble sleeping nearly every night (difficulty falling asleep, waking up in the middle of the night, early morning wakening or sleeping excessively)? |  |  |
| 5. | Did you talk or move more slowly than normal or were you fidgety, restless or having trouble sitting still almost every day? Did anyone notice this? |  |  |
| 6. | Did you feel tired or without energy almost every day? |  |  |
| 7. | Did you feel worthless or guilty almost every day? |  |  |
| 8. | Did you have difficulty concentrating or making decisions almost every day? |  |  |
| 9. | Did you repeatedly think about death, have any thoughts of killing yourself, or have any thoughts of killing yourself, or have any intent or plan to kill yourself? Did you attempt suicide? |  |  |
| 10. | Do these symptoms cause significant distress or problems at home, at work, socially, in your relationships, or in some other way, and are they a change from previous functioning? |  |  |
